# Consumer Perception and Technological Characterization of Mini Cakes Prepared With Special Beers

**DOI:** 10.1111/1750-3841.71188

**Published:** 2026-06-18

**Authors:** Fabiele Nazaré Tavares, Tassyana Vieira Marques Freire, Katiúcia Alves Amorim, Maria Laura Silva Galdino, Luíz Guilherme Malaquias da Silva, Dieyckson Osvani Freire, Ana Carla Marques Pinheiro

**Affiliations:** ^1^ Department of Food Science Federal University of Lavras, DCA/UFLA Lavras Minas Gerais Brazil

**Keywords:** bakery innovation, concept product, sensory evaluation

## Abstract

This study evaluated consumer perception of mini cakes prepared with special beers and developed formulations in which milk was fully replaced by different beer styles. Two complementary studies were conducted. First, a structured online questionnaire was applied to 100 Brazilian consumers to assess perceptions of the product concept. Most participants (94%) considered the beer‐based mini cakes highly innovative and associated beer addition with changes in flavor, texture, color, and caloric content, reporting low reluctance to consume the product. In the second study, five formulations were prepared: a control with milk and four formulations containing Stout, Red Ale, Weiss, or Lager beers. Physical analyses included instrumental color, texture profile, and volume, whereas sensory evaluation was performed with 100 consumers using check‐all‐that‐apply (CATA) and a 9‐point hedonic scale. No significant differences (*p* > 0.05) were observed between beer‐based samples and the control for texture, volume, or color, indicating that milk replacement did not impair technological quality. CATA revealed that the control was associated with “sweet taste” and “moist cake,” Red Ale cakes with “fluffy” and “soft cake,” and Stout cakes with “beer aroma” and “firmness.” “Sweet taste,” “sweet cake aroma,” and “characteristic cake flavor” were positively related to acceptance, whereas “beer flavor” negatively influenced liking. All formulations achieved good consumer acceptance (6.39–6.64), corresponding to “slightly liked” to “moderately liked.” Overall, special beers can be successfully used as alternative ingredients in mini cake formulations, adding sensory differentiation without compromising quality or acceptance.

## 1 Introduction

Bakery products are integral components of diets worldwide, with cakes standing out as particularly appealing items (Guiné 2022). Among the different cake varieties, muffins or mini cakes, an Anglo‐American product, are notable for being individually portioned and widely accepted globally due to their sweet flavor, soft texture, and moist mouthfeel (Martínez‐Cervera et al. 2012).

Mini cakes, given their convenience and sensory appeal, represent potential matrices for incorporating new flavors. However, reformulating and developing novel products requires careful attention to preserving their technological characteristics (Aranibar et al. 2018). The total or partial substitution of certain cake ingredients can improve the sensory quality of the product (Braga‐Souto et al. 2022). Most studies on cakes have focused on replacing flour, fat, or sugar (Alves et al. 2020; Barros et al. 2021; Braga‐Souto et al. 2022). Liquids, in turn, are essential for providing the necessary moisture to the dough, facilitating mixing, and ensuring an appropriate texture. The most commonly used liquids in cake preparation include water, milk (fresh, curdled, or reconstituted milk powder), and fruit juices (Ornellas 1995). Nevertheless, depending on the recipe, other liquids, such as beers, can be incorporated, imparting unique sensory characteristics to cakes.

Recent market indicators highlight a growing opportunity for innovation in baked goods and cakes. The global cake market is projected to expand significantly, being valued at approximately $100.87 billion in 2025 and forecasted to reach $123.45 billion by 2030, with a CAGR of about 4.12% (Mordor Intelligence 2025). This growth is driven by factors, such as the premiumization of artisanal cake lines, the rising acceptance of “free‐from” recipes, and ongoing innovations in flavors, functional ingredients, and packaging. These trends underscore a growing consumer demand for novelty, quality, and health‐conscious bakery products.

Beer, conversely, is among the most recognized fermented beverages worldwide and has a particularly strong presence in Brazil, with production ranging from large‐scale industrial breweries to artisanal operations (Gluger and Gurak 2020). Given these market dynamics, there is a clear demand for products that combine innovation, sensory appeal, and functionality. Although some studies have investigated the incorporation of brewery by‐products into bakery items (Daniel et al. 2018; Shih et al. 2020), there remains a gap regarding the direct replacement of milk with special beers in cakes and its impact on technological and sensory properties.

Beyond technological and sensory considerations, the incorporation of special beers into bakery products also represents a relevant innovation for contemporary gastronomy. The use of fermented beverages as functional and sensory‐modulating ingredients aligns with current gastronomic trends focused on cross‐category ingredient application, flavor complexity, and product differentiation.

In this context, the substitution of milk with special beers in cake formulations can be justified not only as a strategy to introduce novel sensory attributes but also as an approach to explore the technological potential of fermented beverages in bakery products. Beer contains compounds, such as residual sugars, amino acids, and phenolic compounds, which may influence browning reactions, flavor development, and overall product characteristics. Additionally, the replacement of traditional ingredients with alternative components aligns with current industry trends focused on innovation, product differentiation, and the development of value‐added formulations. Similar approaches have been explored in bakery systems, demonstrating that the incorporation of alternative ingredients can be achieved without compromising technological performance while promoting product innovation (Su et al. 2023). Therefore, this study aimed to evaluate consumer perception of the concept of mini cakes formulated with special beers and to develop and characterize formulations prepared with different beer styles as substitutes for milk, analyzing their technological and sensory parameters.

## 2 Materials and Methods

### 2.1 Study Overview

**FIGURE 1:**
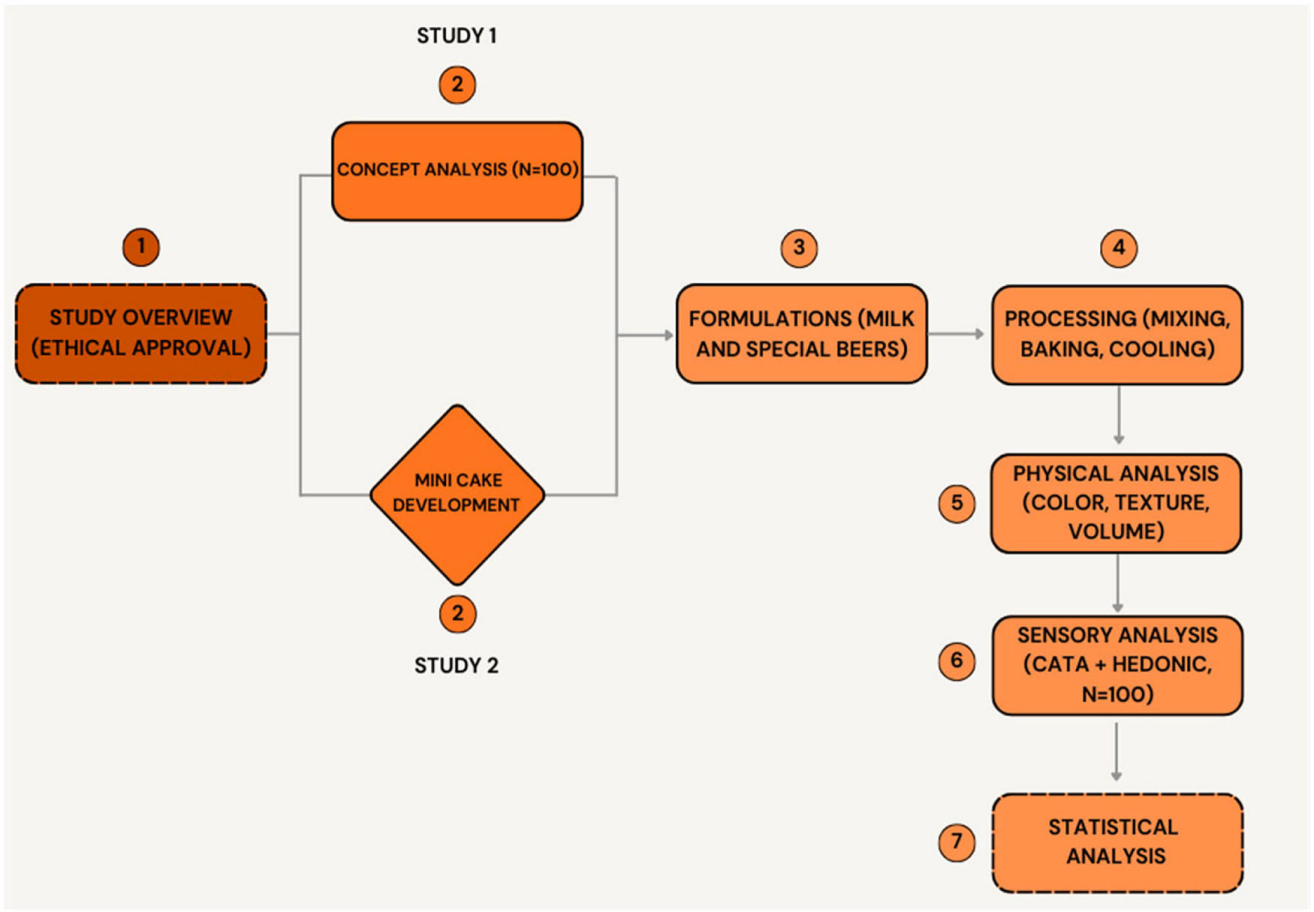
Flowchart of the experimental design, including concept analysis, formulation development, physical characterization, sensory evaluation, and statistical analysis of mini cakes prepared with special beers.

Two complementary studies were conducted. The first consisted of an online questionnaire designed to assess consumer perception of the concept of mini cakes formulated with special beers. The second involved the development and physico‐sensory characterization of mini cakes prepared by fully replacing milk with different beer styles (Stout, Red Ale, Weiss Ale, and Lager), including physical analyses (color, volume, and texture profile) and sensory evaluations (check‐all‐that‐apply [CATA] and overall acceptance test). A flowchart summarizing the experimental design is presented in Figure 1.

### 2.2 Study 1: Concept Analysis

The concept evaluation aimed to identify consumer perceptions of mini cakes made with the addition of special beers, as well as to map potential market niches. For this purpose, a structured questionnaire was developed and administered through the Google Forms platform. The questionnaire consisted of 18 items addressing sociodemographic factors (age, education level, household income, gender, and geographic region), eating habits with a focus on consumption frequency (mini cakes, traditional beers, and special beers), and aspects related to the product concept (degree of innovation, purchase intention, perceived advantages of consuming the mini cake, and agreement scales regarding the influence of beer on attributes, such as flavor, texture, color, and caloric value).

A pilot study with 20 respondents was initially conducted to identify potential issues related to question formulation, omissions, or interpretation difficulties (Mitchell et al. 2012; Rodrigues et al. 2025). The questionnaire was subsequently revised on the basis of participant feedback to ensure clarity, with items that confused being reformulated to improve precision and comprehension. After these adjustments, the final version of the questionnaire was made available online. The sample consisted of 100 Brazilian participants recruited through non‐probabilistic convenience sampling, voluntarily, via invitations distributed through social media and email (Garcia et al. 2025; Rodrigues et al. 2025).

Responses from the online questionnaire were exported from Google Forms and analyzed using descriptive statistics and cross‐tabulations. Summary tables and figures were generated in Microsoft Excel.

### 2.3 Study 2: Development and Characterization of Mini Cakes With Special Beers

#### 2.3.1 Formulations and Preparation

Five mini cake formulations were developed as follows: One control formulation (A), prepared with whole milk, and four formulations in which milk was fully replaced by special beers of the styles Stout (B), Red Ale (C), Weiss Ale (D), and Lager (E). The proportions of ingredients were based on Santos and Boêno (2016) with adaptations defined through preliminary trials, as described in Table 1.

**TABLE 1:** Formulations used for the preparation of mini cakes with full replacement of milk by special beers.

Ingredients	Amount (%)				
	A	B	C	D	E
Wheat flour	30.25	30.25	30.25	30.25	30.25
Brown sugar	24.20	24.20	24.20	24.20	24.20
Soybean oil	10.60	10.60	10.60	10.60	10.60
Milk	15.20	—	—	—	—
Stout beer	—	15.20	—	—	—
Red Ale beer	—	—	15.20	—	—
Weiss (Ale) beer	—	—	—	15.20	—
Lager beer	—	—	—	—	15.20
Baking powder	0.60	0.60	0.60	0.60	0.60
Salt	0.15	0.15	0.15	0.15	0.15
Eggs	19.0	19.0	19.0	19.0	19.0

*Note*: A = control with milk; B = Stout beer; C = Red Ale beer; D = Weiss Ale beer; and E = Lager beer.

All ingredients were purchased from local retail establishments, duly licensed and registered with the sanitary authorities, within their shelf‐life, and bearing complete labeling (Tavares et al. 2022). Processing was carried out at the Laboratory of Sensory Analysis of Food and Beverages, Federal University of Lavras (UFLA).

After weighing the ingredients, the preparation followed six steps, as described by Tavares et al. (2022): (i) whipping the egg whites at high speed for 5 min in an electric mixer to incorporate air; (ii) adding brown sugar and mixing at high speed for 1 min; (iii) incorporating the yolks, milk or beer, and vegetable oil, followed by mixing at medium speed for 2 min; (iv) gradually adding wheat flour and baking powder, previously sifted, and mixing at medium speed for 2 min; (v) pouring the batter into silicone molds (≈35 g of raw dough per unit); and (vi) baking in a preheated electric oven at 170°C for 25 min. After baking, the mini cakes were cooled at room temperature (∼25°C) for 15 min and immediately subjected to physical and sensory analyses.

#### 2.3.2 Physical Characterization

The mini cakes were characterized for volume, instrumental color, and texture profile analysis (TPA). All analyses were performed in quintuplicate.

The apparent volume of the mini cakes was determined using the millet seed displacement method (Queiroz 2001). On the basis of the volume and baked weight, the specific volume (mL g^−1^) was calculated for each formulation (Barros et al. 2018).

Instrumental color was measured using a Color Quest XE colorimeter (HunterLab, USA), according to the CIELAB system. Lightness (L*), red–green chromaticity (a*), and yellow–blue chromaticity (b*) coordinates were recorded as the average of readings taken at different points on the upper surface of the mini cakes. Chroma (C*) and hue angle (*h*°) were calculated (Pathare et al. 2013) as follows: C* = (a*^2^ + b*^2^)^0,5^; *e* *h* = tan^−1^ (b*/a*).

TPA was performed using a TA.XT.PLUS texturometer (Stable Micro Systems, Godalming, England). For TPA, a P/75 probe was used, with pre‐test, test, and post‐test speeds of 1.0 mm s^−1^, and a deformation rate of 40%. For the cutting force (CF) test, a cutting plate was used, with pre‐test and test speeds of 1.0 mm s^−1^, a post‐test speed of 10 mm s^−1^, and a deformation rate of 150%. The texture parameters analyzed included hardness, chewiness, cohesiveness, springiness, adhesiveness, gumminess, and resilience (Braga et al. 2024).

#### 2.3.3 Sensory Analysis

The sensory study was conducted at the Laboratory of Sensory Analysis of Food and Beverages of the Federal University of Lavras (UFLA), in accordance with ISO 8589: 2007, and following the best practices described by Lawless and Heymann (2010). CATA and acceptance tests were applied, according to the methodologies described by Ares and Jaeger (2015) and Amorim et al. (2023). A total of 100 regular cakes, over 18 years of age and of both genders, voluntarily participated in the study. All participants provided informed consent through the informed consent form.

The attributes used in the CATA test were previously defined by 25 consumers using Kelly's repertory grid method (Silas Souza et al. 2024; Garcia et al. 2025). This group was composed of individuals with a heterogeneous demographic profile, including different age ranges (predominantly between 18 and 40 years) and both genders, with a higher proportion of female participants, ensuring variability in perception and vocabulary during attribute elicitation. Participants were recruited from the university community (students and staff) at UFLA.

In this procedure, panelists analyzed the samples in pairs and described the similarities and differences perceived (Moskowitz 2002). The terms generated were subsequently discussed in a group session, aiming to eliminate rarely mentioned descriptors and to retain only those considered most relevant and familiar for characterizing the products. It is important to note that the consumers involved in the initial term selection did not participate in the final CATA evaluation (Garcia et al. 2025).

The CATA questionnaire included 14 descriptors related to appearance (brown crust color, light crumb color, dark crumb color, and golden crust color), aroma (sweet, beer, and characteristic cake), flavor (sweet taste, beer, and characteristic cake), and texture (soft, fluffy, moist, and firm). In the CATA questionnaires, the terms were presented in randomized order for each consumer, using a ballot format that grouped the descriptors by sensory modality (Amorim et al. 2023).

The questionnaire also included a structured 9‐point hedonic scale, ranging from 1 (dislike extremely) to 9 (like extremely) (Stone et al. 2020). Samples were coded with random three‐digit numbers and served on white polystyrene trays, accompanied by a glass of water to cleanse the palate between tastings (Wakeling and MacFie 1995).

#### 2.3.4 Statistical Data Analysis

Physical parameters were evaluated using analysis of variance (ANOVA) followed by Tukey's test to verify differences among samples at a 5% significance level (*p* ≤ 0.05).

For the data obtained from the CATA test, the evaluations were based on the proportion of consumers who selected each term. Responses were organized in a binary format, in which the presence of each attribute was coded as 1 and its absence as 0. This procedure allowed the construction of a contingency table on the basis of selection frequencies, representing how often each attribute was mentioned by the participants. After data screening and validation, 89 responses were considered valid and included in the statistical analysis. Cochran's *Q* test was applied to the binary data to assess significant differences among samples, followed by multiple pairwise comparisons using Sheskin's critical difference procedure at a 5% significance level. Attributes without significant differences were excluded from further interpretation. Correspondence analysis (CA) was performed on the basis of the contingency table using chi‐square distance, and a test of independence was applied to evaluate the association between samples and attributes. Perception maps were also constructed (Meyners et al. 2013; Amorim et al. 2023). Furthermore, penalty‐lift analysis was conducted to quantify the impact of individual sensory attributes on liking scores, identifying attributes that positively or negatively influenced hedonic responses (Garcia et al. 2025; Galdino et al. 2025). All analyses were performed using XLStat 2022 software (Addinsoft, Paris, France). The complete CATA data matrix is provided as Supporting Information.

The data from the structured 9‐point hedonic scale were initially subjected to normality testing (Shapiro–Wilk) and evaluation of ANOVA assumptions. As the data did not meet the normality assumption (*p* < 0.05), parametric analysis was not considered appropriate. Therefore, the data were analyzed using frequency histograms in order to characterize the distribution of consumer responses and to identify acceptance patterns, such as dispersion and possible polarization (Amorim et al. 2023; Passos et al. 2025).

## 3 Results and Discussion

### 3.1 Product Concept

The product concept study was conducted through a structured questionnaire with 100 participants, predominantly female (82.0%) and aged between 18 and 40 years (50.0% between 18 and 30 years and 36.0% between 31 and 40 years). Most participants, resided in the Southeast region of Brazil (88.0%), had completed higher education and reported a household income of up to three minimum wages (61.0%). Regarding consumption frequency, 87.0% of participants reported consuming mini cakes, 79.0% reported consuming traditional beers, and 77.0% reported consuming special beers, albeit rarely or occasionally. A total of 32.0% stated that they consumed mini cakes, and 35.0% reported consuming special beers at least once a week or every 15 days.

According to ABIMAPI (2025), the industrialized biscuits, pasta, bread, and cakes sector started 2025 with a growth trend, recording revenues of R$19 billion between January and April 2025, an increase of 2.7% compared with the same period in 2024. The greatest growth was observed in the industrialized cake category, which reached R$900 million in the first 4 months of the year, representing a 12.5% increase compared to the previous year, along with an 8.8% rise in volume (21 million tons). These figures highlight the growth potential and innovative opportunities in this segment. From an industrial perspective, these findings reinforce that the development of innovative bakery products, such as mini cakes incorporating special beers, aligns with current market trends toward product differentiation and value addition. The positive consumer perception of the concept, combined with the growing demand for premium and artisanal products, suggests that this type of formulation may represent a viable strategy for product diversification in the bakery sector, particularly for niche markets seeking novel sensory experiences (Semenova and Semenov 2023).

It is well established that food choice is a complex process, influenced by multiple factors related to the product (physical, chemical, and sensory characteristics), the individual (age, sex, education, and psychological factors), and the cultural, social, and economic context (Ramya and Alia 2016). In this context, mini cakes prepared with the addition of special beers can be characterized as an innovative bakery product, designed for individual portions and quick consumption.

**FIGURE 2:**
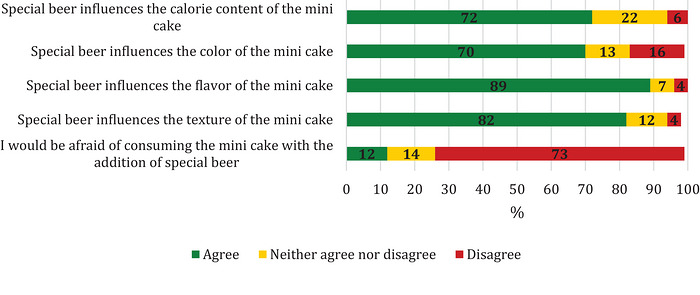
Consumers’ agreement scale regarding the concept of mini cakes with the addition of special beer.

The vast majority of participants (94%) evaluated the concept as highly innovative, assigning a score of 7 or higher on a 9‐point scale. Regarding perceptions of possible modifications induced by beer, most respondents agreed that it could influence the sensory attributes and calorie content of the mini cakes (Figure 2). Specifically, 72% agreed that beer could affect calorie content, 70% indicated an effect on color, 89% on flavor, and 82% on texture. These results demonstrate that consumers clearly associate beer with changes in both the composition and the sensory profile of the product. Despite these perceptions, most respondents stated that they would not be afraid of consuming mini cakes prepared with special beers: 73% disagreed with this statement, whereas only 12% agreed and 14% remained neutral. This result indicates a generally favorable attitude toward trying the product, even when consumers anticipate potential changes in its sensory and nutritional properties.

### 3.2 Mini Cake

#### 3.2.1 Color

The color of the mini cakes was estimated using the Nix sensor, and the data were represented through the online Free Color Converter (Nix Sensor 2025), which provided the mean L*, C*, and h* parameters of the CIELAB system and allowed visual representation in different color spaces, such as HEX, RGB, and CMYK. The obtained values are presented in Table 2.

**TABLE 2:** Instrumental color of mini cakes.

Samples	*L* *	C*	*h* *	Color estimate**
A	40.46 ± 1.81aba	17.48 ± 1.73ab	68.67 ± 4.16a	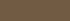
B	39.12 ± 2.40a	16.62 ± 2.19a	67.53 ± 2.55a	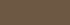
C	41.89 ± 2.45b	19.01 ± 1.86b	70.35 ± 4.79a	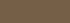
D	39.45 ± 2.34a	16.47 ± 2.33a	66.72 ± 7.87a	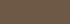
E	39.57 ± 1.74a	17.47 ± 2.42ab	68.71 ± 3.31a	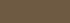

^*^ In each column, different letters represent statistically significant differences at the 0.05 significance level according to Tukey's test.

^**^ Visual color representation according to the Nix Color Sensor, available at https://www.nixsensor.com/free-color-converter/.

The results showed moderate lightness (39.12–41.89), typical of baked products with a darker tone. The chroma (16.47–19.01) indicated low to moderate color saturation, whereas the hue angle (66.72° to 70.35°) corresponded to a yellowish‐reddish hue, which is characteristic of bakery products. Among the formulations, Sample C exhibited significantly higher L* and C* values (*p* < 0.05) compared to Samples A, B, D, and E, evidencing greater brightness and color intensity. No significant differences were observed for the h* parameter, confirming the stability of the predominant hue.

These findings demonstrate that, despite the slight increase in brightness and saturation observed in Sample C, the addition of special beer did not produce relevant changes in the overall color of the mini cakes, ensuring chromatic uniformity among the formulations. Such stability is desirable because color is one of the main quality attributes perceived by consumers, directly influencing expectations of freshness and overall product acceptance (Pathare et al. 2013).

It is also worth noting that raw materials with naturally dark pigmentation, such as certain vegetable flours, cocoa bran, brown sugar, and fermented beverages like special beer, can directly influence the color profile of baked goods by intensifying or darkening their final appearance (Bellini et al. 2022). This effect is related to the presence of phenolic compounds, natural pigments, and browning reactions, such as caramelization and Maillard reactions that occur during thermal processing. In the present study, however, even with the inclusion of special beer, an ingredient with potential to darken the crumb, no significant alterations were detected in the L*, C*, and h* parameters (Sarques et al. 2024). This confirms that the formulation maintained color stability and visual uniformity, an essential attribute for consumer acceptance and for the development of foods with both strong sensory appeal and sustainable value. From a technological standpoint, the maintenance of color stability is particularly relevant for industrial applications, as it ensures product standardization and visual consistency across batches. This is a critical requirement for large‐scale production, where deviations in color may negatively affect consumer perception and brand reliability.

#### 3.2.2 Texture and Volume

According to Teotônio et al. (2021), texture and specific volume are key quality attributes in bakery products, directly influencing consumer acceptance. Table 3 presents the mean values of instrumental texture parameters (hardness, adhesiveness, springiness, cohesiveness, gumminess, chewiness, and resilience) and the volume of mini cakes prepared with different special beers.

**TABLE 3:** Instrumental texture and volume of mini cakes.

Parameters	A	B	C	D	E
Hardness (N)	1204.13 ± 499.84a	1315.96 ± 277.27a	1197.43 ± 256.60a	1065.06 ± 248.95a	1001.60 ± 301.24a
Adhesiveness (N)	−201.45 ± 244.63a	−210.54 ± 192.12a	−360.37 ± 209.17a	−166.79 ± 150.31a	−249.46 ± 153.25a
Springiness (%)	0.83 ± 0.085a	0.87 ± 0.07a	0.84 ± 0.05a	0.85 ± 0.06a	0.83 ± 0.09a
Cohesiveness (%)	0.57 ± 0.05a	0.60 ± 0.09a	0.64 ± 0.04a	0.57 ± 0.13a	0.61 ± 0.09a
Gumminess (N)	709.41 ± 315.52a	807.05 ± 229.86a	777.89 ± 181.55a	627.57 ± 223.62a	634.20 ± 231.05a
Chewiness (N)	602.12 ± 283.74a	702.42 ± 214.62a	655.95 ± 162.83a	546.41 ± 214.01a	533.51 ± 202.13a
Resilience (%)	0.24 ± 0.03a	0.25 ± 0.04a	0.26 ± 0.02a	0.24 ± 0.06a	0.26 ± 0.04a
Volume (g cm^3^)	81.93 ± 15.33a	80 ± 14.70a	83.56 ± 12.70a	80.12 ± 14.17a	92.43 ± 11.33a

*Note*: Different letters within a row indicate statistically significant differences (*p* < 0.05) according to Tukey's test.

Mini cakes formulated with special beers (Stout, Red Ale, Weiss, and Pilsner) showed no significant differences from the control sample for any of the texture or volume parameters. This finding indicates that partially replacing liquid ingredients with special beer did not compromise crumb structure or technological quality. The absence of significant differences in texture parameters can be explained by the structural nature of cake systems. Cake texture is mainly determined by the formation of a viscoelastic matrix involving starch, proteins, and incorporated air during baking. In this context, liquid ingredients are primarily responsible for providing the moisture necessary for batter development, facilitating mixing, and ensuring proper texture (Marzec et al. 2021). In this context, replacing milk with beer did not significantly affect texture, likely because the overall formulation remained balanced, preserving the batter structure and gas retention capacity. Despite the replacement of milk with special beers, the formulation retained key structural ingredients, such as flour, eggs, and sugar, which are primarily responsible for structure formation, gas retention, and crumb development.

In addition, beers contain water, residual sugars, and soluble compounds that can perform similar functional roles in the batter system, contributing to moisture retention and structural stability.

Hardness, which expresses the force required to compress the sample (Barros et al. 2021), ranged from approximately 1001–1316 N without significant differences, confirming that beer addition did not increase firmness. A similar behavior was reported by da Silva et al. (2024) in gluten‐free cookies prepared with cocoa bran (solution of cocoa bran and butter), where the presence of fibers and phenolic compounds did not compromise structural integrity. Adhesiveness, which reflects stickiness and is associated with starch and water release during baking (Gasparre et al. 2019), varied from −166.79 to −360.37 N, with no significant variation.

Springiness, indicating the ability to return to its original shape after deformation, ranged from 0.83% to 0.87%, whereas resilience, which reflects structural recovery, varied from 0.24% to 0.26%, remaining stable across all formulations. Cohesiveness, indicative of internal bonding strength, ranged from 0.57% to 0.64%, likewise unaffected by beer addition. This structural stability is consistent with the findings of Batista et al. (2025), who reported that incorporating ora‐pro‐nobis flour, rich in fiber and bioactive compounds, did not impair the texture of gluten‐free cupcakes.

Gumminess and chewiness, which express the energy required to disintegrate and chew the product (Alves et al. 2020), ranged from 627.57 to 807.05 N and from 533.51 to 702.42 N, respectively, with no significant differences observed. Specific volume, which reflects gas retention and expansion capacity, varied from 80.00 to 92.43 g cm^3^, confirming that the addition of special beers did not affect crumb aeration or development.

These results reinforce that in bakery products, organoleptic properties, particularly texture, are decisive for consumer acceptance, as they encompass physical and sensory characteristics that directly influence the perception of quality and the value attributed to the product. Beyond their sensory impact, textural properties also have technological and economic importance, as they are closely linked to product stability and help reduce losses during processing, storage, and transportation (Guiné 2022).

The absence of significant changes in texture and volume indicates that the incorporation of special beers does not require major modifications in processing conditions or formulation structure. This represents a relevant advantage for the bakery industry, as it enables the development of innovative products without compromising process efficiency, production costs, or product standardization.

### 3.3 Sensory Analysis

Table 4 shows the citation proportions of the attributes used in the characterization of the mini cakes by the CATA test. Significant differences were observed between the control sample and the formulations with the addition of special beers for several attributes.

**TABLE 4:** Proportion of citations of different attributes evaluated in the check‐all‐that‐apply (CATA) test with Cochran's *Q* test, for the assessment of differences in the proportion of citations among samples.

Attributes	A	B	C	D	E
Brown crust color^***^	0.337ab	0.506b	0.258a	0.528b	0.472b
Golden crust color^NS^	0.517a	0.528a	0.449a	0.427a	0.483a
Light crumb color^***^	0.506bc	0.270a	0.629c	0.326ab	0.360ab
Dark crumb color^***^	0.202ab	0.382c	0.135a	0.348bc	0.247abc
Sweet cake aroma^NS^	0.461a	0.326a	0.472a	0.360a	0.472a
Beer aroma^***^	0.135a	0.337b	0.270ab	0.236ab	0.337b
Characteristic cake aroma^NS^	0.551a	0.404a	0.483a	0.472a	0.539a
Sweet taste^***^	0.742b	0.483a	0.596ab	0.562ab	0.584ab
Beer flavor^***^	0.056a	0.404c	0.348bc	0.180ab	0.393c
Characteristic cake flavor^**^	0.652b	0.416a	0.517ab	0.539ab	0.539ab
Soft cake^NS^	0.809a	0.674a	0.798a	0.697a	0.775a
Fluffy cake^NS^	0.730a	0.607a	0.719a	0.607a	0.697a
Moist cake^**^	0.438b	0.225a	0.326ab	0.315ab	0.270ab
Firm^***^	0.258a	0.427b	0.292ab	0.404ab	0.281ab

*Note*: Pairwise comparisons using McNemar's procedure (Bonferroni): different letters within the same row indicate significant differences. A—control mini cake prepared with milk; B—mini cake prepared with Stout beer; C—mini cake prepared with Red Ale beer; D—mini cake prepared with Weiss (Ale) beer; E—mini cake prepared with Lager beer.

^**^Indicates significant differences among samples according to Cochran's *Q* test, with *p* ≤ 0.01.

^***^Indicates significant differences among samples according to Cochran's *Q* test, with *p* ≤ 0.001.

Regarding color attributes, the control sample (A; 0.337) did not differ from the others for “brown crust color,” whereas Sample C recorded the lowest frequency (0.258), differing from the other beer‐based formulations. For “light crumb color,” Sample B presented the lowest frequency (0.270), differing from Samples C (0.629) and A (0.506). Sample C was the most associated with this attribute, whereas Sample A did not differ significantly from C, D, or E. For “dark crumb color,” Sample C showed the lowest frequency (0.135), differing from Samples B (0.382) and D (0.348). Sample B also differed from Sample A (0.202), which, in turn, did not differ from C, D, or E.

For aroma attributes, only “beer aroma” showed significant differences. Sample A registered the lowest frequency (0.135), differing from Samples B (0.337) and E (0.337), which presented the highest values. Samples C (0.270) and D (0.236) did not differ significantly from each other or from the other samples.

With respect to flavor attributes, for “sweet taste,” Sample A recorded the highest frequency (0.742), differing from Sample B (0.483), which had the lowest. Samples C (0.596), D (0.562), and E (0.584) did not differ significantly from each other or from the others. For “beer flavor,” Sample A presented the lowest frequency (0.056), differing from Samples B (0.404), C (0.348), and E (0.393), which did not differ from each other. Sample D (0.180) did not differ significantly from either A or C. For “characteristic cake flavor,” Sample A showed the highest frequency (0.652), differing from Sample B (0.416), which had the lowest. Samples C (0.517), D (0.539), and E (0.539) did not differ significantly from each other or from the remaining samples.

Regarding texture attributes, for “moist cake,” Sample A presented the highest frequency (0.438), differing from Sample B (0.225), which recorded the lowest. Samples C (0.326), D (0.315), and E (0.270) did not differ significantly from each other or from the others. For “firm,” Sample B showed the highest frequency (0.427), differing from Sample A (0.258). Samples C (0.292), D (0.404), and E (0.281) did not differ significantly from the other samples.

**FIGURE 3:**
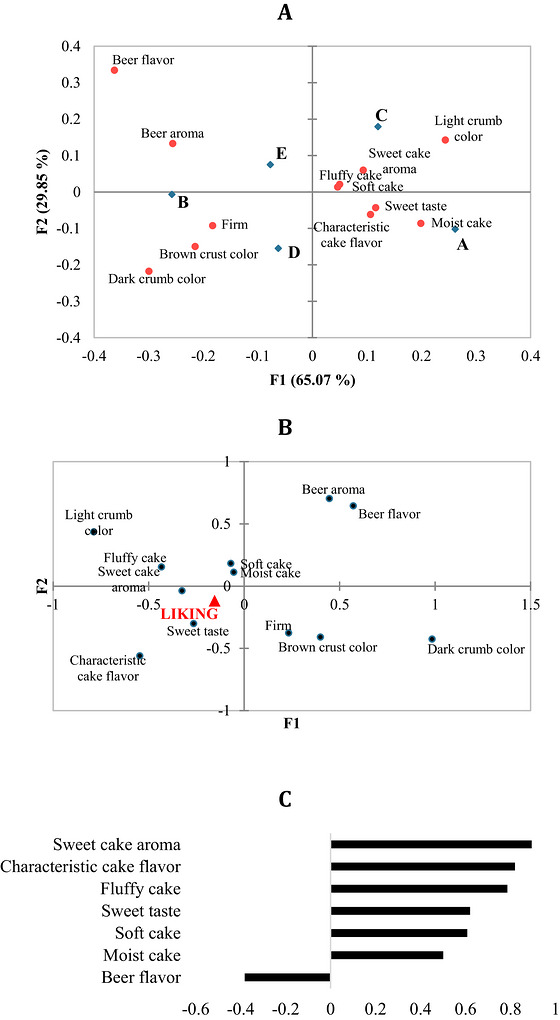
Correspondence analysis (A), principal coordinates analysis (B), and penalty‐lift analysis (C) of sensory attributes of mini cakes with special beers. A = control with milk; B = Stout beer; C = Red Ale beer; D = Weiss Ale beer; E = Lager beer.

Figure 3 presents the results of the CA, principal coordinates analysis, and penalty‐lift analysis of the sensory attributes of mini cakes with special beers. The CA (Figure 3A), obtained from the contingency table of CATA data, explained 94.9% of the total variability. This approach allows the visualization of the associations between the mini cake formulations and the sensory attributes cited by consumers. The mini cake prepared with Red Ale beer was mainly associated with the attributes “fluffy cake,” “soft cake,” “light crumb color,” and “sweet cake aroma.” The formulation with Stout beer was related to the attributes “firm,” “brown crust color,” and “beer aroma.” The formulation control sample was associated with “sweet taste” and “moist cake.”

The results of the principal coordinate analysis (Figure 3B) highlighted the sensory attributes most related to consumer acceptance of the mini cakes. “Sweet taste,” “sweet cake aroma,” and “characteristic cake flavor” were the main descriptors positively associated with sample acceptability. To a lesser extent, but still contributing positively, were “fluffy cake,” “soft cake,” and “moist cake.” Notably, these attributes largely correspond to the characteristics of the standard formulation already marketed, whereas the attributes associated with the addition of special beers were not decisive for consumer acceptance.

Figure 3C shows the results of the penalty‐lift analysis, which evaluated the influence of specific sensory attributes on product acceptance. Among the evaluated attributes, “sweet cake aroma,” “characteristic cake flavor,” “fluffy cake,” “sweet taste,” “soft cake,” and “moist cake” had a positive effect on acceptance. In contrast, the attribute “beer flavor” negatively impacted product acceptance.

Taken together, these results provide important insights into how consumers perceive mini cakes prepared with special beers. The results reinforce that fluffy and soft texture, as well as sweet taste, are key attributes for the acceptance of mini cakes, corroborating previous studies that highlight the importance of these factors in the quality of cakes and bakery products (Martínez‐Cervera et al. 2012). In the present study, these attributes were more strongly associated with the control formulation prepared with milk, which explains their positive contribution to overall acceptability. Milk, in addition to enriching flavor and aroma, acts as an emulsifier, improves color, and promotes dough stability, directly influencing texture and the uniformity of the internal structure (Eleutério et al. 2014). The complete replacement of milk with special beers, although conferring differentiated sensory characteristics, led to greater prominence of attributes, such as “beer flavor” and “beer aroma,” which negatively impacted acceptance. This finding suggests that, for cakes, consumers tend to value traditional attributes related to softness, sweetness, and characteristic cake flavor, whereas sensory notes associated with beer were not decisive in driving consumer preference.

It should be noted that the present study did not include an evaluation of nutritional composition or health implications associated with replacing milk with beer, as its scope was limited to technological and sensory aspects. Therefore, no conclusions can be drawn regarding the relative healthiness of the formulations. However, the complete replacement of milk may represent a potential alternative for consumers with lactose intolerance or those following lactose‐restricted diets, without implying an overall nutritional advantage.

These findings have important implications for product development in the bakery industry. They suggest that the incorporation of beer should be carefully optimized to enhance product differentiation without compromising key drivers of consumer acceptance, such as sweetness, softness, and characteristic cake flavor (Shakhman et al. 2025). Partial substitution strategies or the use of beer as a flavor‐enhancing component, rather than a complete replacement of traditional ingredients, such as milk, may represent a more effective approach to balance innovation and acceptability. From a gastronomic perspective, these results indicate that special beers can be used as complementary ingredients to enhance aromatic and flavor complexity in baked products, provided that their sensory intensity is carefully balanced. The preservation of texture and volume supports the feasibility of incorporating fermented beverages into both artisanal and professional baking. Moreover, the differentiated sensory profiles associated with each beer style offer opportunities for menu innovation, pairing strategies, and storytelling in contemporary gastronomy. Thus, beer‐based mini cakes may be explored not only as novel food products, but also as creative gastronomic elements. Further studies are warranted to evaluate the nutritional composition and potential health implications of this substitution.

The overall acceptance scores of the mini cakes ranged from 6.39 to 6.64 on the 9‐point hedonic scale, with no significant differences among formulations (*p* > 0.05). The control sample prepared with milk obtained a mean score of 6.64 ± 1.58, whereas the beer‐based formulations showed similar results: Red Ale (6.58 ± 1.55), Lager (6.56 ± 1.89), Weiss (6.48 ± 1.57), and Stout (6.39 ± 1.66). These values indicate that all samples were rated between “slightly liked” and “moderately liked,” demonstrating good consumer acceptance.

**FIGURE 4:**
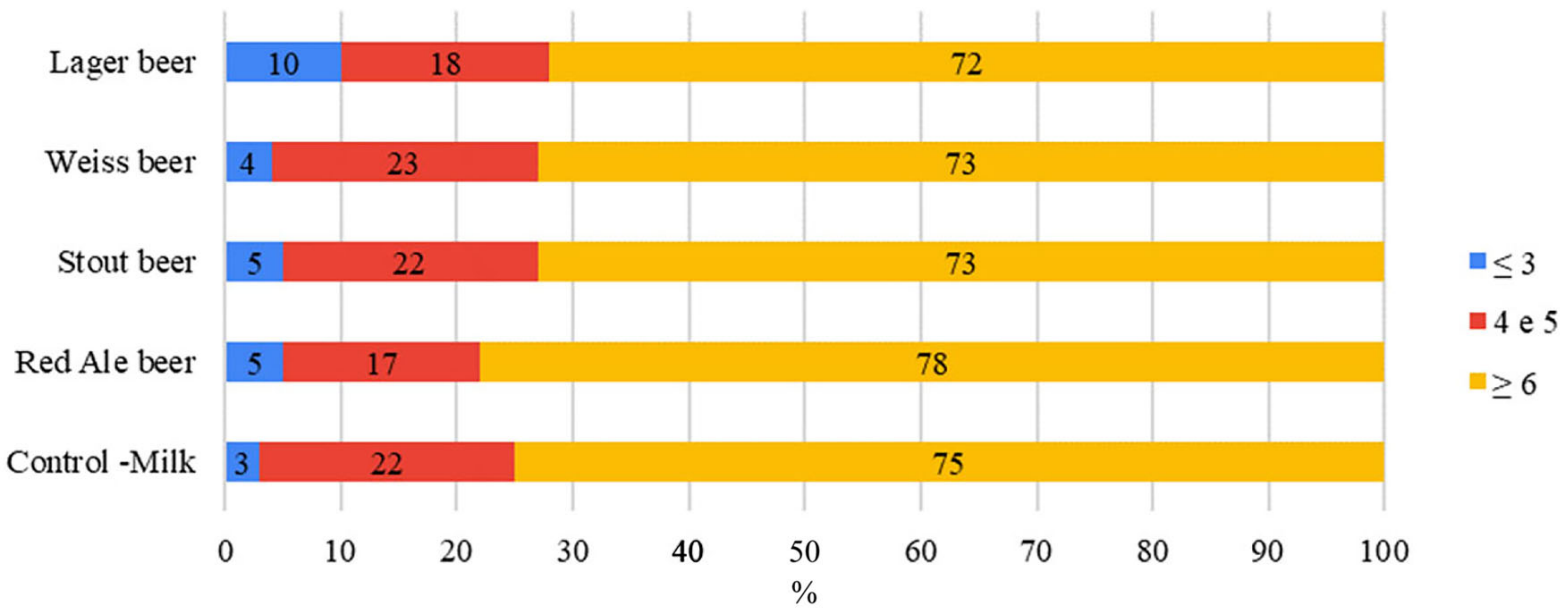
Frequency distribution of consumer acceptance scores for mini cakes.

Figure 4 shows the distribution of consumer responses across the hedonic scale categories. The majority of panelists (72%–78%) rated the samples with scores ≥6, whereas only a small proportion (3%–10%) gave ratings ≤3.

Previous studies have also explored the incorporation of ingredients derived from brewing processes into bakery products. Daniel et al. (2018) developed and sensorially evaluated cookies formulated with by‐products from craft brewing and reported good consumer acceptance and purchase intention. Similarly, Shih et al. (2020) evaluated the quality and consumer acceptance of muffins fortified with spent grain flour from Ale brewing and observed that muffins containing 15% of this ingredient were well accepted and even perceived as more attractive than traditional formulations. These findings suggest that brewing‐derived ingredients can be successfully incorporated into bakery products. In the present study, the direct substitution of milk with special beers did not negatively affect consumer acceptance, as all samples reached comparable hedonic scores. Nevertheless, consumers clearly perceived beer‐related attributes, such as “beer flavor” and “beer aroma,” which distinguished the beer‐based formulations from the control. This indicates that although overall liking was maintained, the sensory profile of the mini cakes was significantly modified by the addition of beer.

Overall, the results of this study demonstrate that the incorporation of special beers into mini cakes is technologically feasible and does not negatively impact key quality attributes, such as texture, volume, and overall acceptance. However, sensory optimization remains essential, particularly regarding the control of beer‐related flavor notes. From an industrial perspective, these findings support the potential application of special beers as innovative ingredients in bakery products, contributing to product differentiation, market expansion, and the development of value‐added formulations aligned with current consumer trends.

## 4 Conclusion

This study demonstrated that mini cakes formulated with special beers constitute an innovative bakery product that meets consumer expectations while maintaining technological and sensory quality. The addition of Stout, Red Ale, Weiss, or Lager beers did not significantly affect instrumental color, texture, or specific volume, ensuring visual stability and structural integrity of the crumb. Sensory analysis confirmed overall acceptance comparable to the control formulation, with traditional cake attributes, such as sweetness, softness, and characteristic flavor remaining the main drivers of consumer preference, even when beer‐related notes were perceptible. These findings confirm that the replacement of milk with special beers allows for product diversification without compromising quality or consumer acceptance, fulfilling the study's objective of developing a differentiated bakery product with market potential and offering new opportunities for creative applications in contemporary gastronomy.

## Author Contributions

**Fabiele Nazaré Tavares**: conceptualization, investigation, writing – original draft, methodology, formal analysis. **Tassyana Vieira Marques Freire**: investigation, writing – original draft, visualization, formal analysis. **Katiúcia Alves Amorim**: data curation, formal analysis, methodology, validation, investigation, writing – original draft, writing – review and editing. **Maria Laura Silva Galdino**: investigation, writing – original draft, visualization, formal analysis, methodology. **Luíz Guilherme Malaquias da Silva**: investigation, writing – original draft, visualization, formal analysis, data curation. **Dieyckson Osvani Freire**: supervision, writing – original draft, writing – review and editing. **Ana Carla Marques Pinheiro**: writing – review and editing, visualization, validation, project administration, supervision, conceptualization, methodology.

## Ethics Statement

This study was approved by the Research Ethics Committee involving Human Subjects at the Federal University of Lavras (Approval No. 5.174.007). All participants provided informed consent prior to participation.

## Conflicts of Interest

The authors declare no conflicts of interest.

## Supporting information




**Supplementary materials**: jfds71188‐sup‐0001‐SuppMat.xlsx

## Data Availability

All data generated or analyzed during this study are included in this published article and are available from the corresponding author on reasonable request.
